# Lowbush Wild Blueberries have the Potential to Modify Gut Microbiota and Xenobiotic Metabolism in the Rat Colon

**DOI:** 10.1371/journal.pone.0067497

**Published:** 2013-06-28

**Authors:** Alison Lacombe, Robert W. Li, Dorothy Klimis-Zacas, Aleksandra S. Kristo, Shravani Tadepalli, Emily Krauss, Ryan Young, Vivian C. H. Wu

**Affiliations:** 1 Department of Food Science and Human Nutrition, The University of Maine, Orono, Maine, United States of America; 2 United States Department of Agriculture ARS, BARC, Bovine Functional Genomics Laboratory, Beltsville, Maryland, United States of America; 3 Department of Molecular and Biomedical Sciences, The University of Maine, Orono, Maine, United States of America; Cairo University, Egypt

## Abstract

The gastrointestinal tract is populated by an array of microbial species that play an important role in metabolic and immune functions. The composition of microorganisms is influenced by the components of the host’s diet and can impact health. In the present study, dietary enrichment of lowbush wild blueberries (LWB) was examined to determine their effect on colon microbial composition and their potential in promoting gut health. The microbial composition and functional potential of the colon microbiota from Sprague Dawley rats fed control diets (AIN93) and LWB-enriched diets (AIN93+8% LWB powder substituting for dextrose) for 6 weeks were assessed using Illumina shotgun sequencing and bioinformatics tools. Our analysis revealed an alteration in the relative abundance of 3 phyla and 22 genera as representing approximately 14 and 8% of all phyla and genera identified, respectively. The LWB-enriched diet resulted in a significant reduction in the relative abundance of the genera *Lactobacillus* and *Enterococcus*. In addition, hierarchal analysis revealed a significant increase in the relative abundance of the phylum Actinobacteria, the order Actinomycetales, and several novel genera under the family Bifidobacteriaceae and Coriobacteriaceae, in the LWB group. Functional annotation of the shotgun sequences suggested that approximately 9% of the 4709 Kyoto Encyclopaedia of Gene and Genome (KEGG) hits identified were impacted by the LWB-diet. Open Reading Frames (ORFs) assigned to KEGG category xenobiotics biodegradation and metabolism were significantly greater in the LWB-enriched diet compared to the control and included the pathway for benzoate degradation [PATH:ko00362] and glycosaminoglycan degradation [PATH:ko00531]. Moreover, the number of ORFs assigned to the bacterial invasion of epithelial cells [PATH:ko05100] pathway was approximately 8 fold lower in the LWB group compared to controls. This study demonstrated that LWBs have the potential to promote gut health and can aid in the development of optimal diets.

## Introduction

Dietary enrichment of blueberries demonstrated impacts on gut microbial population dynamics and gastro-intestinal tract (GI) health [1]–[3]. Lowbush wild blueberries (*Vaccinium angustifolium*) (LWB) are excellent sources of fiber, manganese, and polyphenols, such as anthocyanins [4]. Research on functional foods that can promote gut health and beneficial microbiota has become a topic of interest in the field of preventive medicine, and there is paucity of research as to their potential impacts on gut microbial ecosystems. The modulation of the gut microbiota in response to diet has been further linked to different components of metabolic syndromes such as obesity, inflammation, insulin resistance, and type-2 diabetes [5], [6]. Lowbush wild blueberries have demonstrated several health benefits, including attenuation of indicators of metabolic syndrome and inflammation, although little is known of role of gut flora in this process [5]–[7].

Microbial catabolic reactions can yield products that are more biologically active than the parent compounds, and therefore may be more beneficial to the host [8]–[10]. In the colon, nutrients are processed through a complex and diverse bioreactor consisting of 10^9^−10^12^ microorganisms, and may contribute up to a 10% increase in caloric absorption [11]. In the large intestine, polyphenols are transformed by the intestinal microbiota before being absorbed; these reactions have been previously characterized [8], [12], [13]. In humans, the intestinal absorption of dietary polyphenols is often slow and largely incomplete, and up to 85% of lowbush blueberry anthocyanins enter the colon intact, depending on moiety and glycosylation pattern [14]. Several metabolic pathways were proposed for the catabolism of phenolic acids, anthocyanins, and proanthocyanidins by the intestinal microbiota [15], [16]. Different metabolic pathways for the digestion of polyphenols could be attributed to variations in the microbiota composition, and different pathways could coexist, depending on the catabolic capacity of the microbiota [17].

Lowbush wild blueberries have demonstrated a positive effect on native GI microbiota through the increase of beneficial microorganisms [3], [16], [18]. In humans, dietary treatment with LWB increased the population of *Bifidobacteria* more than two fold [3], suggesting LWB’s prebiotic activity. In rats, a blueberry-enriched diet attenuated the symptoms of intestinal colitis and reduced fecal *Enterobacteriaceae* counts [19]. In ulcerative colitis patients, members of the *Enterobacteriaceae* family and different *Enterococcus* species have been documented to increase with a concomitant decrease in *Bifidobacteria* [20], [21]. It is possible that dietary interventions with LWB may help ameliorate inflammation by altering the microbial composition in favor of *Bifidobacteria* and other beneficial species. Recent developments in molecular biology allow for the simultaneous analysis of genes and have revealed insights into the molecular basis of the native gut microbiota in response to dietary changes. Advances in DNA sequencing technology have dramatically changed the way scientists investigate the microbial communities that populate the gut [22]. Metagenomic studies have enormous potential and can be utilized to investigate the microbial function in response to dietary alterations by describing the functional genomics of microbial communities and their potential physiological phenotypes.

There is a lack of knowledge with respect to how LWB affect the function and cellular mechanics of gut microbes and how this interaction could potentially impart health benefits to the host. The objectives of this research were to i) characterize the function of the gut microbial community of the Sprague Dawley (SD) rats using metagenomic techniques and ii) use this model to detect compositional and functional changes in a LWB enriched diet. Investigating changes in gut microbiota in response to LWB enrichment may provide insights into how LWBs impart health benefits to their host.

## Methods

### Animal Experiment

The study was carried out in strict accordance with the recommendations in the Guide for the Care and Use of Laboratory Animals of the National Institutes of Health. All animal procedures were approved by the Institutional Animal Care and Use Committees of the University of Maine (Protocol #A2011-01-03). Nine male, three-week old Sprague-Dawley (SD) rats, approximately 90 g in weight, were obtained from Charles River Laboratories (Wilmington, MA). They were randomly divided into 2 groups: the control group (N = 4), which was fed a control diet (AIN93), and the treatment group (N = 5), which was fed a blueberry-enriched diet, (AIN93+8% w/w LWB powder substituting for dextrose) [23]. The supplier analyzed the LWB powder and microbial load was below the detection threshold (1 log). Chemical analysis demonstrated that the total ACN content of the LWB powder was 1.5% w/w and contained 21 different ACNs, primarily malvidin 3- galactoside and peonidin 3-galactoside [7]. Tap water and diet were provided *ad libitum* and diet consumption was measured daily. Animals consumed 20±4 g of feed per day, an amount equivalent to 24.0±5.2 mg of anthocyanins and 4.5 g of fiber per day in the LWB diet [9]. After 6 weeks of feeding, the animals were asphyxiated using 95% CO_2_/5% O_2_ inhalation for 3 min and samples from the large intestine were taken as follows.

### Sampling and DNA Extraction

Animals were dissected in a laminar flow hood to ensure sterility. Once the rat cavity was fully exposed, the intestines were unwound. Samples for metagenomic analysis were taken from the proximal colon, approximately one inch downstream of the cecum. The colon contents (0.37±0.13 g) were collected by elevating one end of the large intestine and pushing contents into a sterile sample container. The samples were then snap-frozen in liquid nitrogen and stored at −80°C. A QIAamp DNA stool kit was used to extract metagenomic DNA(Qiagen, Valenica, CA) with modifications to the protocol described by Li *et al*., 2011 [24]. Lysis incubation at 95°C for 6 min was used to replace the 70°C lysis recommended in the standard protocol. DNA integrity was verified using a BioAnalyzer 2100 (Agilent, Palo Alto, CA). Metagenomic DNA concentration was quantified by fluorometry.

### Metagenomic Sequencing and Analysis

Approximately 1.0 µg of high-quality DNA was processed using an Illumina TruSeq DNA sample prep kit following the manufacturer’s instruction (Illumina, San Diego, CA). Final individual libraries were validated, pooled based on their respective 6-bp adaptors, and sequenced at 100 bp/sequence read using an Illumina HiSeq 2000 sequencer. All Illumina raw sequences were deposited into MG-RAST databases (MG-RAST Accession # from 4470921.3 to 4471270.3). The mean numbers of whole genome shotgun (WGS) sequence reads used in this study are listed in Table 1. After all of the DNA was sequenced, reads from the WGS approach were first trimmed using SolexaQA, a Perl-based software package calculating quality statistics from FASTQ files generated by Illumina sequencers [25]. Quality control filters were applied to WGS raw reads before further analysis. Reads of host origin were then removed using Bowtie, an ultrafast memory-efficient short read aligner [26]. The remainder of WGS sequences was *de novo* assembled using SOAP*denovo* software package (http://soap.genomics.org.cn/soapdenovo.html), and normalized to ensure that each genome was represented equally regardless of size. Quality reads were then analyzed using MetaPhyler, mapping genes against a reference genomes using BLAST [27]. Bit scores and sequence read lengths were applied to linear regression model at each taxonomic level. The relative abundance data from MetaPhyler were analyzed based on a modified *t*-test [28].

**Table 1:** Summary of metagenomic samples and sequencing results.

	Control (*N* = 4)	LWB (*N* = 5)
**original sequence count**	5.1×10^7^±7.9×10^6^	5.2×10^7^±1.2×10^7^
**after-trimming count**	4.0×10^7^±6.6×10^6^	4.1×10^7^±1.3×10^7^
**after-filtering sequence count**	2.4×10^7^±3.9×10^6^	3.6×10^7^±1.2×10^7^
**after-trimming average length (bp)**	91.7±3.5	89.6±3.5
**after-filtering average length (bp)**	91.5±3.3	89.4±3.5
**N50**	766.2±278	1139.7±743.7
**length of contigs (bp)**	565.8±85	635±155
**assembled length (Mb)**	113.2±22.6	82.1±12.5

Original sequence length 100 bp; LWB = Lowbush wild blueberry.

Raw sequence reads were uploaded into a MG-RAST server for quantitative views of the microbial populations in the rat proximal colon based on WGS sequence data [26]. The data was then analyzed following the MG-RAST pipeline (v3.0) (metagenomics.anl.gov), including quality filtering, dereplication to remove possible sequencing artefacts, and removal of host contaminants. Open reading frames (ORF) were then predicted using FragGeneScan from the remaining reads [29]. The lowest common ancestor method was used for microbial classification in the pipeline. Raw sequence counts positively assigned to a given taxon at the phylum-, class-, family-, and genus- levels were normalized. The relative abundance (% composition) was then square root transformed and analyzed using Primer (v6) (primer-e.com) for the Bray-Curtis similarity matrix. Functional annotation of unassembled shotgun sequences was performed using MG-RAST (v3.0). Additionally, the shotgun sequences were de novo assembled using SOAP*denovo*. Open reading frames (ORFs) were predicted from contigs ≥200 bp using FragGeneScan (v1.14). The resultant ORFs were independently annotated according to the Kyoto Encyclopedia of Genes and Genomes (KEGG) database.

### Statistical Analysis

Statistical analysis was performed using *Metastats (* http://metastats.cbcb.umd.edu). The method relied on two assumptions: first, that each of two treatment populations has multiple samples: and second, that the relative abundance data were available for specific features, such as relative abundance assigned to each taxon, or numbers of sequences mapped to a given biological pathway. The matrix was normalized from raw abundance measures to a fraction representing the relative contribution of each feature to each of the individuals. A modified *t*-test was then used to compute a two-sample *t* statistic. The threshold for the *t* statistic was chosen to minimize the number of false positives by controlling *p* values using the nonparametric *t*-test with 1000 permutations, which dealt with the underlying distributions that were non-normal. For low frequency (sparse) features, such as low abundance taxa, Fisher’s exact test was used to model the sampling process according to a hypergeometric distribution (sampling without replacement).

## Results

### Composition of the Rat Colon Microbiota

**Figure 1:**
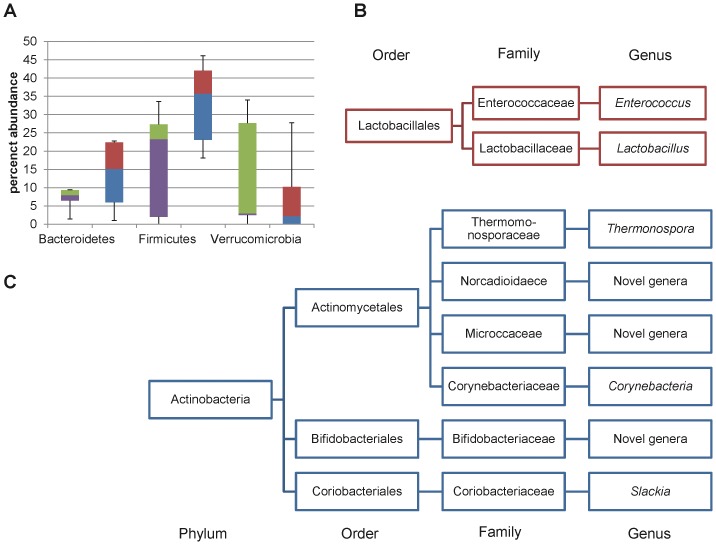
MetaPhyler analysis of the rat proximal colon. A) Most abundant phylum identified. Boxes denote the inner quartile range between the 1^st^ and 3^rd^ quartiles (25% purple = LWB, blue = control; 75% green = LWB, red = control). B) Microorganisms identified at a significantly (*P*<0.05) lower relative abundance. C) Microorganisms identified at a significantly (*P*<0.05) higher relative abundance. LWB = lowbush wild blueberries.

**Figure 2:**
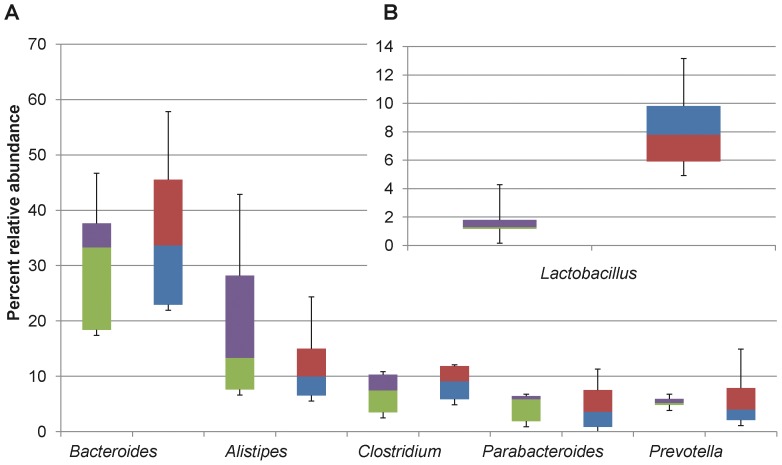
MG-RAST analysis of the rat proximal colon. A) Most abundant genera identified. Boxes denote the inner quartile range between the 1^st^ and 3^rd^ quartiles (25% purple = LWB, blue = control; 75% green = LWB, red = control). B) Significantly (*P*<0.05) impacted genera. LWB = lowbush wild blueberries.

The results from metagenomic sequencing and assembly are summarized in Table 1. Twenty-four prokaryotic phyla were identified in the rat proximal colon using MetaPhyler. Firmicutes, Bacteroidetes, Actinobacteria, and Verrucomicrobia were the dominant phyla in the control rat (Figure 1), which is consistent with mammalian gut microbiome [30]. Among the 59 classes identified, Bacteroidia (44.26% control; 44.25% LWB), Clostridia (32.23% control; 28.44% LWB), and Verrucomicrobaie (1.21% control; 13.72% LWB) were dominant in the rat colon fed a control diet. One hundred and ninety five families were represented, in which Lactobacillaceae (13.90% control; 3.03% LWB; *P* = 0.016), Bacteroidaceae (12.95% control; 9.31 LWB), Rikenellaceae (10.48% control; 19.66% LWB), and Ruminococcaceae (10.17% control; 11.33% LWB) were among the most abundant. Similarly, 306 prokaryotic genera were identified in the rat proximal colon using MetaPhyler. Among them, *Lactobacillus, Bacteroide*s, *Alistipes, Clostridium*, *Ruminococcus*, and *Akkermansia* were the major representatives in the rat colon. In addition to MetaPhyler, the microbial composition of the rat colon microbiota was also characterized independently using the MG-RAST pipeline which identified 32 phyla and 752 genera. The most abundant phyla identified by MG-RAST were Bacteroides, Firmicutes, and Proteobacteria, and the most abundant genera identified were *Bacteriodes*, *Alistipes*, *Clostridium*, *Lactobacillus, Prevotella*, and *Parabacteriodes* (Figure 2).

### Impact of the LWB Diet on the Rat Proximal Colon Microbiota

MetaPhyler and MG-RAST detected significant differences in microbial composition at the genera level (Figure 1 and 2). The homology-based MG-RAST identified fewer taxa than MetaPhyler, which uses a hierarchical approach, allowing for the identification of novel genera. MetaPhyler detected significant changes in the relative abundance of 22 genera between control and LWB diets. While MG-RAST did not detect significant differences in the relative abundance of any phyla, there was a significant reduction of 6.0% in the genus *Lactobacillus* (Figure 2).

Metaphyler detected a significant change in the microbial composition of the rat colon in the LWB treatment group. At the phylum level, Actinobacteria was detected at a significantly (*P*<0.01) 2-fold higher abundance in the LWB rats than the control. Members of the order Actinomycetales were twice as abundant and included the genera *Thermonospora* and *Cornynebacterium*, and novel genera classified under the family *Nocardioidaceae*, *Pseudonocardiaceae*, *Micrococcaceae*, and *Actinomycetaceae* (Table 2). The relative abundance of the family Coriobacteriaceae was detected at a 2.7-fold higher abundance in the LWB diet, in addition to members of that family such as *Slackia* and novel genera. Members of the family Bifidobacteriaceae were detected at 2-fold higher abundance and novel genera under that family were detected in a 5-fold higher abundance in the LWB enriched diet.

**Table 2:** Genera significantly impacted by lowbush wild blueberry (LWB) diet as detected by MetaPhyler.

Genus	Control	LWB		Known attribute
***Lactobacillus***	14.18±3.68×10^−2^		3.08±1.30×10^−2^	Probiotic [19]
Actinomycetales (order)*	0.24±2.95×10^−4^	0.52±7.24×10^−4^		Peroxidase activity [49]
Coriobacteriaceae (family)*	0.10±3.73×10^−4^	0.34±7.41×10^−^4		Hepatic liver function [45]
***Enterococcus***	0.06±1.90×10^−4^	0.00±2.02×10^−5^		Antibiotic resistance [50]
***Slackia***	0.05±1.33×10^−4^	0.11±2.44×10^−4^		Conversion of isoflavones [51]
Myxococcaceae (family)*	0.04±8.62×10^−5^	0.09±1.61×10^−4^		Cancer and antibiotic drug synthesis [52]
Myxococcales (order)*	0.03±1.27×10^−4^	0.08±1.58×10^−4^		Cancer and antibiotic drug synthesis [52]
Nocardioidaceae (family)*	0.03±7.57×10^−5^	0.11±1.62×10^−4^		Soil bacteria [53]
Paenibacillaceae (family)*	0.02±6.35×10^−5^	0.00±2.47×10^−5^		Soil bacterium [54]
Chlorobiaceae (family)*	0.02±4.83×10^−5^	0.00±2.02×10^−5^		Sulfur reduction [55]
Peptococcaceae (family)*	0.02±2.53×10^−5^	0.06±1.54×10^−4^		Microbial Fuel Cells [56]
Chroococcales (order)*	0.01±6.52×10^−5^	0.05±1.13×10^−4^		Sulfur reduction [57]
***Anaeromyxobacter***	0.01±4.12×10^−5^	0.05±1.04×10^−4^		Bioremediation [58]
***Micrococcaceae (family)***	0.01±4.12×10^−5^	0.06±2.03×10^−4^		Normal skin flora, photo protection [59]
***Pseudonocardiacea (family)***	0.00±2.91×10^−5^	0.06±1.64×10^−4^		Bioremediation [60]
***Bifidobacteriaceae (family)***	0.00±2.91×10^−5^	0.02±8.21×10^−5^		Bacteriocin production/Probiotic [61]
***Thermomonospora***	0.00±0.00	0.02±6.85×10^−5^		Degradation of xenobiotics [62]
***Corynebacterium***	0.00±0.00	0.01±4.04×10^−4^		Production of amino acids and nucleotides [63]

Numbers denote the mean relative abundance (%), **±** indicates standard error of the mean, *P*-value was calculated using unpaired *t*-test between LWB (*N* = 5) and control diets (*N* = 4). *Indicates a novel genus within the family or a potential novel genus within the order. LWB = Lowbush wild blueberry.

*Lactobacillu*s was the most abundant genus in the colon microbiota of control rats, accounting for 14.18% of all sequence reads (Table 2). Compared to the control, treatment of a LWB enriched diet significantly reduced its relative abundance from 14.18% to 3.08% (*P*<0.02), which represents almost a 5 fold reduction. In addition, the relative abundance of *Enterococcus* was significantly reduced from 0.06% to 0.002%, representing a 30-fold reduction, as a result of the LWB dietary treatment (Table 2).

### LWB Dietary Enrichment Effects on Microbial Functional Potential in the Rat Proximal Colon

**Figure 3:**
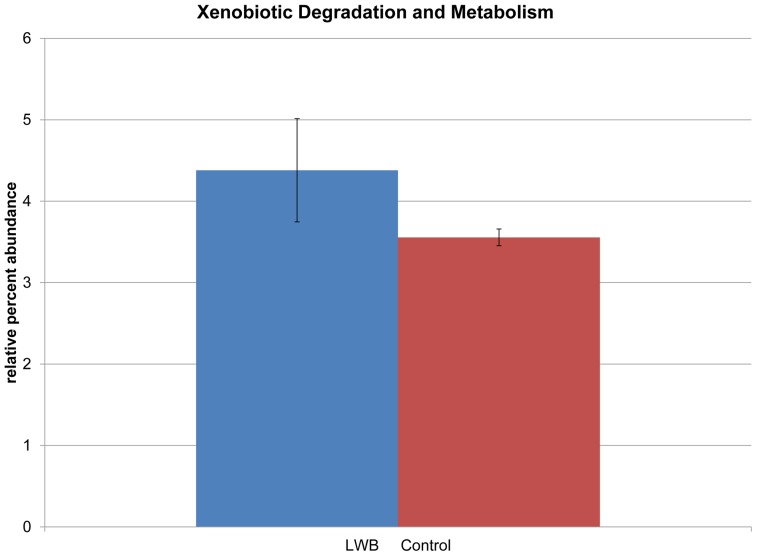
Xenobiotic biodegradation and metabolism was significantly impacted by the lowbush wild blueberries (LWB) diet.

Both assembly-dependent and independent approaches were used to gain insight into the possible changes in the metabolic potentials of the proximal colon in response to the LWB dietary enrichment. Proteins predicted from unassembled DNA sequences were assigned to 278 pathways and 4709 KEGG ontologies (KO) using the MG-RAST pipeline. Statistical comparisons revealed significant changes in ≈9% of all KO identified as a result of the dietary treatment. The five most abundant level 2 categories identified were translation, amino acid metabolism, carbohydrate metabolism, nucleotide metabolism, and replication and repair (Table 3). Among the 37 level-2 KEGG categories, xenobiotic degradation and metabolism had 20% higher abundance in the LWB group (*P*<0.05) (Figure 3). Benzoate degradation [PATH: ko00362], a pathway within xenobiotic degradation and metabolism, was detected at twice the relative abundance in the LWB enriched diet. Similarly, the numbers of ORFs assigned to the pathways such as glycosaminoglycan degradation [PATH: ko00531] and isoleucine/valine degradation [PATH: ko00280], were significantly greater in the LWB treatment group. In addition, the number of ORFs assigned to bacterial invasion of epithelial cells [PATH:ko05100] was approximately 8 fold lower in the LWB group compared to control. KEGG functional annotation using assembled contigs yielded similar results. Notably, the number of ORFs associated with 2, 4 dienoyl –reductase was significantly greater, while the numbers of ORFs annotated to integrase/recombinase, reverse transcriptase, and transposon/transposase were significantly less as a result of LWB treatment.

**Table 3:** Select KEGG pathways significantly impacted by the LWB diet.

Pathway	Pathway ID	Control	LWB
**valine, leucine and isoleucine degradation**	ko00280	1.48	1.89
**glycosaminoglycan degradation**	ko00531	0.01	0.22
**benzoate degradation**	ko00362	0.06	0.15
**phosphotranferase system (PTS)**	ko02060	0.73	0.13
**glycerolipid metabolism**	ko00561	0.04	0.01
**bacterial invasion of epithelial cells**	ko05100	0.02	0.00
**porphyrin and chlorophyll metabolism**	ko00860	0.02	0.00

Numbers denote the mean relative abundance (%) and *P*-value was calculated using unpaired *t*-test between lowbush wild blueberry (LWB) (*N* = 5) and control diets (*N* = 4).

## Discussion

Diet has an influence in shaping the development and composition of the gut microbiota, which is also defined by host genetics and the bacteria acquired at birth [31]. Changes in diet composition can affect the microbial composition and catabolic processes. However, the microbial role in the metabolism of ingested material is not well understood. Recent evidence suggests that gut microbial metabolism has a strong potential for both the bioactivation of essential nutrients and detoxification [14], [32], [33]. Botanical treatments for GI disorders have been used to modify imbalances within the normal physiological boundaries, and therefore the consumption of LWB and its contribution to improving GI health is a subject of considerable interest [3], [34], [35].

The permeability of the colonic epithelium is an important aspect of gastro-intestinal health, and increases in permeability can allow for gut-derived bacteria and toxins to infiltrate the liver via the portal circulation. Blueberries have demonstrated reduction in the degree of parenchymal infiltration and *Enterococcus* and *Clostridium* spp. translocations to the liver in SD rats [21]. The present study observed an 8-fold decrease in the number of ORFs assigned to bacterial invasion of epithelial cells in the LWB diet, possibly corroborating evidence of reduced microbial translocation. *Enteococcus faecalis*, a commensal bacterium of humans and animals, has shown the capability to induce irritable bowel syndrome (IBS) in IL-10 gene-deficient mice, suggesting that certain enteric microbiota tend to be more opportunistic and can induce colon inflammation [36]. Recent research has demonstrated a significant reduction in *Enterococcus* spp. in mice fed diets supplemented with blueberries [37]. Similarly, the present study demonstrated lower levels of *Enterococcus* spp. in the LWB enriched diet. The results from this study and recent research suggest that the protective anti-inflammatory effect of blueberries can be accredited to microbial metabolism, which is dependent on the composition of the microbiota.

Although many microbial members of the large intestine have not been identified, it is known that some species of bacteria preferentially colonize the large intestine [22]. *Bifidobacteria* and *Lactobacilli* are normally present in the large intestine of healthy humans in numbers ranging from 10^8^–10^10^ CFU/ml and 10^6^–10^8^ CFU/ml, respectively [37]. These bacterial species survive in the colon through their ability to degrade and utilize a diverse range of carbohydrates and other compounds using various catabolic enzymes [37]. The present data demonstrates that *Lactobacillus* is higher in the dextrose diets, reflecting their capability to competitively ferment simple sugars in the rat colon. The shifts toward members of the Actinobacteria phylum at the expense of *Lactobacillus* species could be a reflection of the complexity of LWB compound catabolism and that microbial digestion of LWB may require a concerted effort of multiple species [38]. *In vitro* human fecal batch cultures have demonstrated the enrichment of *Lactobacillus* and *Bifidobacteria*, with the addition of 1 g/L gallic acid and 200 mg/L of anthocyanins [39]. In addition, recent studies using the SD rat model fed polyphenols extracted from blueberry via gavage demonstrated an increase in *Lactobacillus* and *Bifidobacteria* [1], [2]. However, *in vivo* studies with humans fed freeze-dried LWB in a smoothie demonstrated significant increases of only *Bifidobacteria* species and no impact on *Lactobacillus* populations [3]. The discrepancies between these studies may be an indication of the differences in native gut microbiota between humans and murine models and/or different fiber and polyphenolic concentrations amongst the diets [3]. Even though dietary models are difficult to standardize and results are difficult to compare, the present study and previous research are in agreement that LWB has prebiotic potential.

Lowbush wild blueberries are exposed to numerous microorganisms with extensive metabolic capacities that are lacking in the host. Previous research demonstrated that anthocyanidins glycosides are hydrolyzed extensively by the intestinal microflora, and the product of microbial digestion is often less stable and possibly more bioavailable [1], [8]. Enzymatic biotransformations of LWB may also be relevant for xenobiotic metabolism, which may allow the conversion of many classes of compounds, including flavonoids, isoflavonoids, lignans, phenolic acids, fiber, and tannins [40], [41]. The present study observed a 20% increase in xenobiotic degradation and, specifically, a two-fold increase in benzoate degradation of SD rats fed a LWB diet. The order Actinomycetales covers a broad group of microorganisms that are well-known for their applications in production of antibiotics and antioxidants for pharmaceuticals, production of numerous enzymes for biocatalysts, and in natural processes such as carbon cycling [42]. Members of this order, namely *Thermospora*, *Corynebacterium*, *Microccaceae*, and *Nocardiodaceae*, were detected in a higher abundance in the LWB enriched diet. In termite hind guts, members of the phylum Acntinobacteria have demonstrated their involvement in xenobiotic metabolism and these microorganisms could possibly contribute to the degradation of benzoate compounds derived from blueberries [42]. Individual bacterial strains belonging to the genera *Bifidobacterium*, *Escherichia*, and *Lactobacillus* isolated from human fecal samples have been reported to execute the hydrolysis reactions of phenolic acids and polyphenols [43]. The genome sequence of *Bifidobacterium longum* has a large number of predicted proteins (more than 8%) related to the catabolism of nondigestible plant polymers, including enzymes involved in the degradation of complex polysaccharides and xenobiotics [43]. The results from this study may demonstrate the benefits of LWB and its impact on bacterial communities with unique functional repertoires by promoting their growth in competitive environments.

Lowbush wild blueberry enrichment may favor microbial species that can harvest energy from LWB components and could possibly promote beneficial microbial populations. Health properties attributed to beneficial bacteria include modulation of colonic microbiota by inhibiting a wide range of pathogens, improvement of lactose digestion, reduction of serum cholesterol, stimulation of the immune system through cytokine stimulus, reinforcement of intestinal epithelial cell tight junctions, and increased mucus secretion [40]. The present study demonstrates that the addition of LWB to diet can alter the balance of gut microbe in favor of members of the Actinobacteria phylum. The Actinobacteria phylum have known impacts on human health, namely, *Slackia*, *Bifidobacteria,* and *Coriobacteriaceae spp*. Research using SD rats demonstrated that blueberry and blackcurrant enriched diets increased *Bifidobacteria* species after dietary treatment and lowered concentrations of pro-inflammatory markers [1]–[3]. In humans, the enrichment of *Bifidobacteria* fed LWB was observed [3] in addition to decreased DNA damage in lymphocytes [44]. Recent studies revealed strong associations of the *Coriobacteriaceae* family with respect to hepatic triglycerides, glucose/glycogen levels, and enterohepatic circulation [45]. The ratio of *Bifidobacterium*/*Coriobacteriaceae* impacted plasma cholesterol levels in hamsters, with *Bifidobacteria* being associated with high density lipoprotein particles (HDL) and *Coriobacteria* being associated with non-HDL particles [46]. Human infants fed breast milk had a higher *Bifidobacterium*/*Coriobacteriaceae* ratio in the intestines than infants fed formula, suggesting a beneficial evolutionary adaptation for the harvest of energy from otherwise indigestible oligosaccharides [47]. Results from the present study demonstrated that the LWB diet produced a higher *Bifidobacterium*/*Coriobacteriaceae* ratio than the control diet. Our results provide insight into microbial community’s response to LWB and should help elucidate the potential benefits in the gut and host.

### Conclusion

Although the microbiome of rats differs from humans, the murine model is a powerful tool to study population dynamics and related metabolic functions. Metagenomic studies can determine microbial community profiles, gene presence/absence and abundance, and functional repertoire; however, they can only infer an observed phenotype since a gene’s presence does not imply its expression or functionality [48]. Consequently, these studies are limited and require integrating multiple layers of information, including transcriptomic, proteomic, and metabolomic data. Understanding the nutrient-microbiome interactions will aid in substantiating health claims about the perceived health benefits of LWBs. Although the direct link between gut microbial function and LWBs perceived health effects has not been concretely established, this information may be used to design diet interventions that aid in promoting gut health and homeostasis.
